# Nano-titanium dioxide inhalation exposure during gestation drives redox dysregulation and vascular dysfunction across generations

**DOI:** 10.1186/s12989-022-00457-y

**Published:** 2022-03-09

**Authors:** Elizabeth C. Bowdridge, Evan DeVallance, Krista L. Garner, Julie A. Griffith, Kallie Schafner, Madison Seaman, Kevin J. Engels, Kimberley Wix, Thomas P. Batchelor, William T. Goldsmith, Salik Hussain, Timothy R. Nurkiewicz

**Affiliations:** 1 Department of Physiology and Pharmacology, 64 Medical Center Drive, Robert C. Byrd Health Sciences Center, West Virginia University School of Medicine, West Virginia University, Morgantown, WV 26505-9229 USA; 2 Center for Inhalation Toxicology, West Virginia University School of Medicine, Morgantown, WV USA

**Keywords:** Engineered nanomaterials, Titanium dioxide, Microcirculation, Kisspeptin, Placenta

## Abstract

**Background:**

Pregnancy is associated with many rapid biological adaptations that support healthy development of the growing fetus. One of which is critical to fetal health and development is the coordination between maternal liver derived substrates and vascular delivery. This crucial adaptation can be potentially derailed by inhalation of toxicants. Engineered nanomaterials (ENM) are commonly used in household and industrial products as well as in medicinal applications. As such, the potential risk of exposure remains a concern, especially during pregnancy. We have previously reported that ENM inhalation leads to upregulation in the production of oxidative species. Therefore, we aimed to determine if F0 dam maternal nano-TiO_2_ inhalation exposure (exclusively) resulted in altered H_2_O_2_ production capacity and changes in downstream redox pathways in the F0 dams and subsequent F1 pups. Additionally, we investigated whether this persisted into adulthood within the F1 generation and how this impacted F1 gestational outcomes and F2 fetal health and development. We hypothesized that maternal nano-TiO_2_ inhalation exposure during gestation in the F0 dams would result in upregulated H_2_O_2_ production in the F0 dams as well as her F1 offspring. Additionally, this toxicological insult would result in gestational vascular dysfunction in the F1 dams yielding smaller F2 generation pups.

**Results:**

Our results indicate upregulation of hepatic H_2_O_2_ production capacity in F0 dams, F1 offspring at 8 weeks and F1 females at gestational day 20. H_2_O_2_ production capacity was accompanied by a twofold increase in phosphorylation of the redox sensitive transcription factor NF-κB. In cell culture, naïve hepatocytes exposed to F1-nano-TiO_2_ plasma increased H_2_O_2_ production. Overnight exposure of these hepatocytes to F1 plasma increased H_2_O_2_ production capacity in a partially NF-κB dependent manner. Pregnant F1- nano-TiO_2_ females exhibited estrogen disruption (12.12 ± 3.1 pg/ml vs. 29.81 ± 8.8 pg/ml sham-control) and vascular dysfunction similar to their directly exposed mothers. F1-nano-TiO_2_ uterine artery H_2_O_2_ production capacity was also elevated twofold. Dysfunctional gestational outcomes in the F1-nano-TiO_2_ dams resulted in smaller F1 (10.22 ± 0.6 pups vs. sham-controls 12.71 ± 0.96 pups) and F2 pups (4.93 ± 0.47 g vs. 5.78 ± 0.09 g sham-control pups), and fewer F1 male pups (4.38 ± 0.3 pups vs. 6.83 ± 0.84 sham-control pups).

**Conclusion:**

In conclusion, this manuscript provides critical evidence of redox dysregulation across generations following maternal ENM inhalation. Furthermore, dysfunctional gestational outcomes are observed in the F1-nano-TiO_2_ generation and impact the development of F2 offspring. In total, this data provides strong initial evidence that maternal ENM exposure has robust biological impacts that persists in at least two generations.

**Supplementary Information:**

The online version contains supplementary material available at 10.1186/s12989-022-00457-y.

## Introduction

A myriad of anatomical and physiological adaptations must take place throughout gestation for a successful pregnancy to occur. Hepatic and vascular adaptation and growth coordinate substrate availability and delivery to the developing fetus [1–3]. This complex, highly regulated, maternal circuit is sensitive to potential toxicant disruption. In 2013, the National Institute of Environmental Health Sciences (NIEHS), formally acknowledged the rise in incidences of adverse pregnancy outcomes associated with exposure to endocrine disrupting chemicals [4]. Even though only the mother is being directly exposed, her offspring and their reproductive capacity may be affected by this gestational insult. The basis for such adult disease can originate from inadequacies of blood flow/substrate delivery during development, which may create a hostile gestational environment and ultimately lead to utero-placental insufficiency. Originally developed by Barker, this theory is recognized as the developmental origins of health and disease (DOHaD) [5, 6]. Inhalation exposure during gestation has the potential to lead to multi- and transgenerational effects. During a toxicological insult, the mother is directly exposed, but the F1 generation experiences a hostile gestational environment during fetal development, and the F2 generation is also subject to this insult as the developing germ cell line with the F1 offspring. If negative health consequences persist into the F3 generation, without subsequent exposure, this is considered a transgenerational effect because these offspring have never been exposed to particles or a hostile gestational environmental associated with the original F0 insult [7]. Nano-TiO_2_ has been shown to cross the placenta [8] during gestational exposure and potentially deposits into the pup [9], however perturbations in vasoreactivity within the uterus of the dam and/or the placenta of the pup alone are enough to result in detrimental fetal health consequences [10].

Engineered nanomaterials (ENM) are defined as materials with at least one dimension less than 100 nm and unique physiochemical properties [11]. The potential toxicity of ENM in biological systems remains an area of specific interest to the National Toxicology Program [12]. Many variables influence the toxicokinetics of a given chemical all of which ultimately focus on dose. We have previously established that a calculated lung deposition of ~ 30 µg per exposure is associated with a ~ 50% impairment in normal arteriolar function [13]. While many factors such as absorption, distribution, and clearance influence toxicity we focused on dose, specifically deposition in this study. Our previous work demonstrated that inhalation exposure to an ENM, such as nano-titanium dioxide (nano-TiO_2_), during gestation disrupts normal uterine [14] and pup [15] arteriolar function [14], placental hemodynamics [16] fetal pup weight, and placental efficiency [10]. However, scant evidence exists as to the underlying mechanisms of these findings and the systemic impact on the subsequent offspring.

Recently, we reported that maternal nano-TiO_2_ inhalation during gestation can result in endocrine disruption, specifically estrogen [10]. During gestation, the peptide hormone kisspeptin (Kiss) [17–19], is associated with hypertension, preeclampsia, and intrauterine growth restriction (IUGR) [20]. We found an augmented Kiss-induced vasoconstriction in uterine arteries of nano-TiO_2_ exposed dams [10]. Interestingly, circulating Kiss levels were not different despite significantly decreased estrogen levels in this study [10]. Hypothalamically, low levels of estrogen are inhibitory to Kiss production whereas higher estrogen levels stimulate Kiss [21]. It is possible that circulating or local levels of estrogen are permissive to Kiss vasoreactivity in the uterine vasculature. This strongly suggests alterations in converging or competing cellular pathways account for the differential Kiss responses following nano-TiO_2_ inhalation exposure.

Redox signaling pathways are critical in maintaining cellular homeostasis and dysregulation of these pathways contribute to several pathologies [22–25]. Hydrogen peroxide is one of the major reactive signaling molecules. Upregulation of H_2_O_2_-mediated production is linked to NF-κB-mediated signaling [26, 27], which is known to mediate both vascular and hepatic dysfunction [28, 29]. It has been reported that pulmonary exposure and estrogen deficiency lead to localized and systemic changes in cellular redox signaling [30–34], but the consequences of these perturbations during gestation on maternal and fetal health are vastly understudied. Redox signaling mediated by H_2_O_2_-regulation of signaling factors can influence multiple hepatic and vascular processes [35]. Interestingly, Kiss has been shown to contribute to the antioxidant defense system within the porcine follicle [22–29, 36]. Whether maternal nano-TiO_2_ inhalation disrupts H_2_O_2_-mediated signaling in critical tissues during pregnancy or whether redox signaling is altered in the F1 generation is unexplored.

Our previous work demonstrates maternal ENM inhalation exposure during gestation perturbs uterine vascular function [10]. Thus setting our rationale for investigating nano-TiO_2_-mediated impacts on the subsequent offspring, based on the DOHaD theory. Therefore, the purpose of this study was to investigate redox signaling in both mother and offspring and determine if the previously defined Kiss response was present during pregnancy in the F1 generation. We hypothesized that inhalation exposure during gestation in the F0 dams leads to vascular, and redox dysfunction that propagates to the F1 generation, and results in smaller F2 pups.

## Results

### Experiment 1

#### Characterization of nano-TiO_2_

**Fig. 1:**
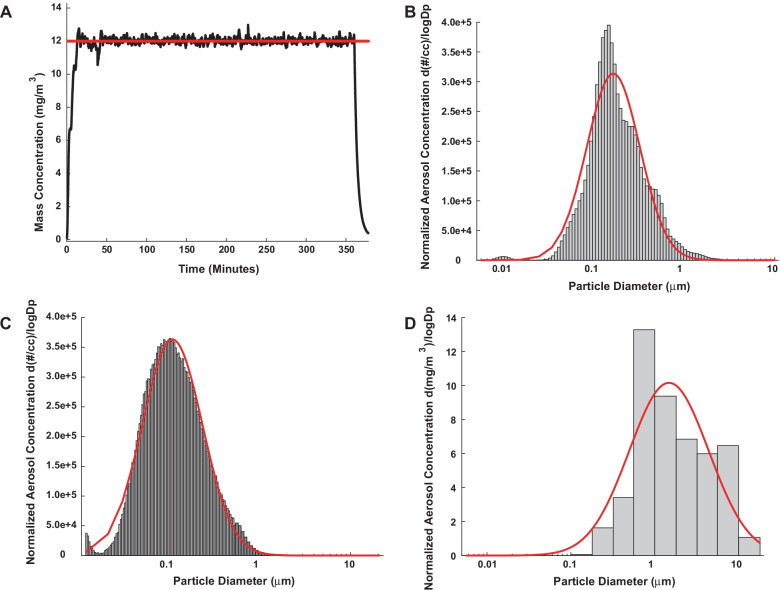
Characterization of nano-TiO_2_. **A** Representative real-time mass concentration measurements of the nano-TiO_2_ aerosol during a typical inhalation exposure. The red line represents the target concentration, 12.35 ± 0.13 mg/m^3^. **B** Size distribution of the nano-TiO_2_ aerosol (aerodynamic diameter) using a high-resolution electrical low-pressure impactor (ELPI+). The red line represents a log-normal fit of the histogram (count median diameter = 170) and geometric standard deviation (GSD) or 1.95. **C** Size distribution of the nano-TiO_2_ aerosol (mobility diameter) sampled from the exposure chamber using a scanning mobility particle sizer (SMPS—light gray) and an aerodynamic particle sizer (APS—dark gray, negligible values). The red line is representative of a log-normal fit of the histogram (count median diameter = 116 nm) with a GSD or 2.12. **D** Mass size distribution of TiO_2_ aerosols sampled from the exposure chamber with a Nano Micro-Orifice Uniform Deposit Impactor (MOUDI 115R, MSP Corp, Shoreview, MN). The red line represents a log normal fit of the data that indicates a mass median aerodynamic diameter (MMAD) of 1.48 µm with a GSD of 2.98

The nano-TiO_2_ aerosol concentration was determined to be 12.35 ± 0.13 mg/m^3^ (Fig. 1A). The ELPI High Resolution data indicated a Geometric Count Median Diameter of 170 nm with a Geometric Standard Deviation of 1.95 (Fig. 1B). The SMPS and APS data were combined to determine the Geometric Count Median Diameter using the log normal distribution obtained with the log probability plot method and was determined to be 116 nm with a Geometric Standard Deviation of 2.12 (Fig. 1C). Figure 1D is the mass size distribution of nano-TiO_2_ aerosols sampled from the exposure chamber with a Nano Micro-Orifice Uniform Deposit Impactor (MOUDI 115R, MSP Corp, Shoreview, MN). The mass median aerodynamic diameter of 1.48 µm with a geometric standard deviation of 2.98.

#### Hepatic and placental redox activity of F0 dams and F1 progeny, and F1 litter characteristics

##### F0 maternal liver characteristics

**Fig. 2:**
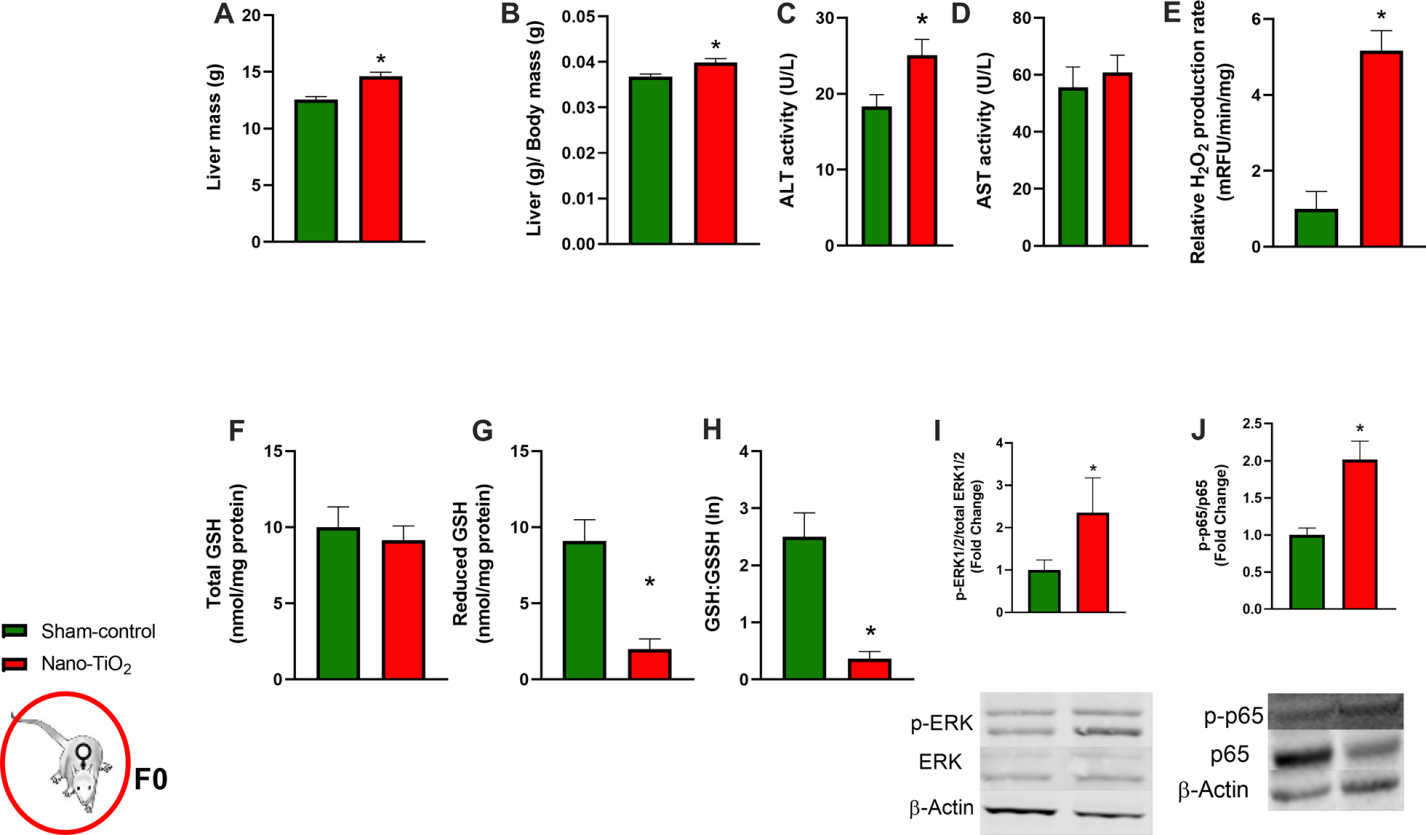
F0 dam liver characteristics. **A** F0 Maternal liver mass and **B** normalized liver mass at GD 20. **C** Coumarin boronic acid detection of H_2_O_2_ accompanied by detection and calculation of **D** total GSH **E** reduced GSH and **F** GSH:GSSH ratio in maternal liver homogenate. **G**, **H** Western blot detection of the phospho/total ratio of ERK 1/2 and the p65 subunit of NF-κB. n = 3–10. *p < 0.05 Sham-control group versus nano-TiO_2_-exposed groups

It is well documented that liver mass increases in proportion to body weight gain during pregnancy. Comparing maternal liver mass at GD 20, nano-TiO_2_ exposed mothers’ livers were significantly greater than those of sham-control mothers, even after normalization to body weight (Fig. 2A, B). Additionally, ALT but not AST was elevated at GD 20, nano-TiO_2_ exposed mothers (Fig. 2C, D). We then analyzed H2O2 production capacity in maternal livers and found nano-TiO_2_ exposed maternal livers exhibit roughly sixfold higher H_2_O_2_ production capacity (Fig. 2E). Concomitantly, lower levels of reduced GSH and a diminished GSH:GSSH ratio were found (Fig. 2F–H). Supporting this elevated H_2_O_2_ signaling we found elevated activation of ERK and p-p65 as a marker of NF-κB (Fig. 2I–J). Together, this suggests redox homeostasis/signaling is altered in the maternal liver following nano-TiO_2_ inhalation exposure. This is an impactful observation due to the critical role the maternal liver plays in fetal substrate regulation.

##### F1 litter characteristics

**Fig. 3:**
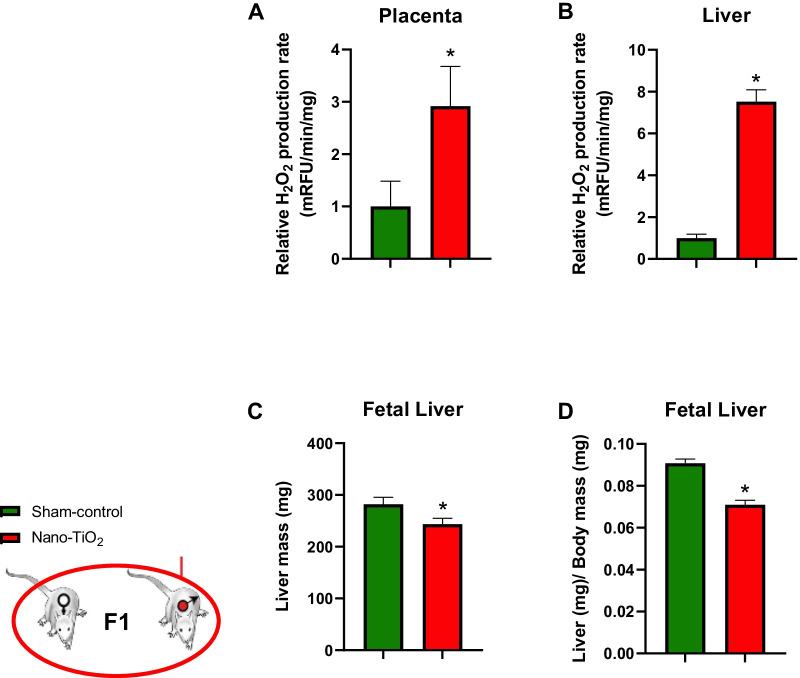
Fetal liver and placental characteristics from F1 litters. **A** H_2_O_2_ production capacity detected by coumarin boronic acid in F1 placenta and **B** liver homogenates at GD 20. **C** F1 fetal liver mass and **D** normalized liver mass at GD 20. n = 5–39; *p < 0.05 Sham-control group versus nano-TiO_2_-exposed groups

F1 litter size was significantly reduced when F0 dams were exposed to nano-TiO_2_ (10.22 ± 0.6 pups) during gestation compared to sham-controls (12.71 ± 0.96 pups; Table 1). Additionally, fewer male pups were born in nano-TiO_2_ litters (4.38 ± 0.3 pups vs. 6.83 ± 0.84 pups exposed vs. sham-control, respectively). Nano-TiO_2_ dams also displayed an increased number of pup resorptions, which was indicated by significantly more implantation sites compared to pups born alive (13.6 ± 1.27 spots vs. 10.22 ± 0.6 pups) compared to sham-controls (12.25 ± 0.79 spots vs. 12.71 ± 0.96 pups; *p* < 0.05; Table 1). Placental tissue displayed greater H_2_O_2_ production capacity in the nano-TiO_2_ exposed group compared to sham-control (Fig. 3A). Similar to maternal livers, fetal livers of mothers exposed to nano-TiO_2_ exhibit greater H_2_O_2_ production capacity (Fig. 3B). We previously reported at GD 20 pups of mothers exposed to nano-TiO_2_ were significantly smaller [10]. Consistent with this previous observation nano-TiO_2_ pups also exhibited less liver mass (Fig. 3C). However, livers from nano-TiO_2_ pups were significantly small even after normalization to body weight on GD 20 (Fig. 3D). Therefore, we can conclude that nano-TiO_2_ inhalation exposure during gestation results in decreased litter size, potentially through causing resorption of fetuses, and skews the sex ratio of the offspring such that males seem to be more greatly affected by maternal ENM exposure. Considered together, nano-TiO_2_ inhalation exposure alters hepatic mass and induces redox dysfunction in dams as well as their progeny.

**Table 1:**
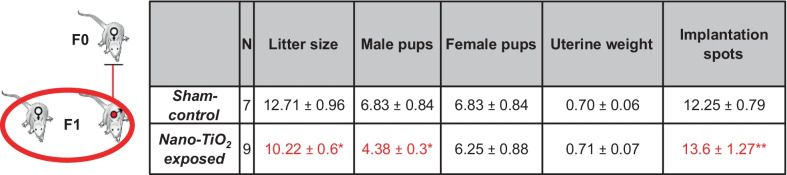
Litter characteristics from F0 dams

Dam and litter characteristics in sham-control (N = 7) and nano-TiO_2_ inhalation exposed (N = 9) groups. Values are shown as mean ± SEM.

*p < 0.05 Sham-control group versus nano-TiO_2_-exposed groups. **p < 0.05 Litter size versus implantation spots in nano-TiO_2_-exposed group.

##### F1 systemic redox dysfunction

**Fig. 4:**
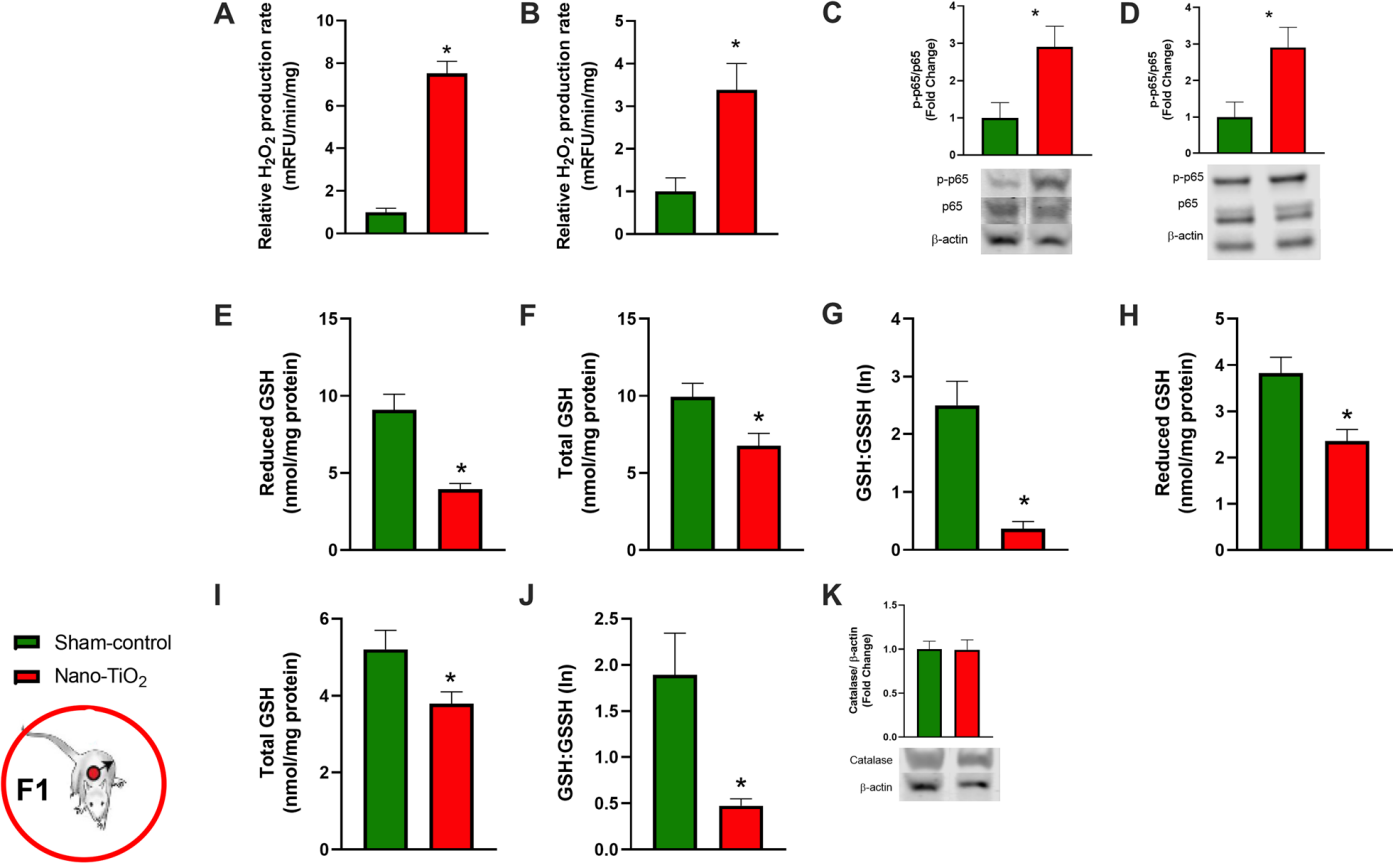
Hydrogen peroxide production rate in tissues from F1 males at 8 weeks of age. F1 plasma samples analyzed for **A** IL-6, **B** glucose, and **C** protein carbonyl content. **C** H_2_O_2_ production capacity detected by coumarin boronic acid in liver homogenates with assessment of liver glutathione **D** reduced GSH, **E** total GSH, and **F** GSSH:GSH ratio and **G**, **H** western blot detection of catalase and phospho NF-κB. **I** H_2_O_2_ production capacity detected by coumarin boronic acid in aorta homogenates with assessment of liver glutathione **J** reduced GSH, **K** total GSH, and **L** GSSH:GSH ratio and **M** western blot detection of phospho NF-κB. n = 4–20; *p < 0.05 Sham-control group versus nano-TiO_2_-exposed groups

**Fig. 5:**
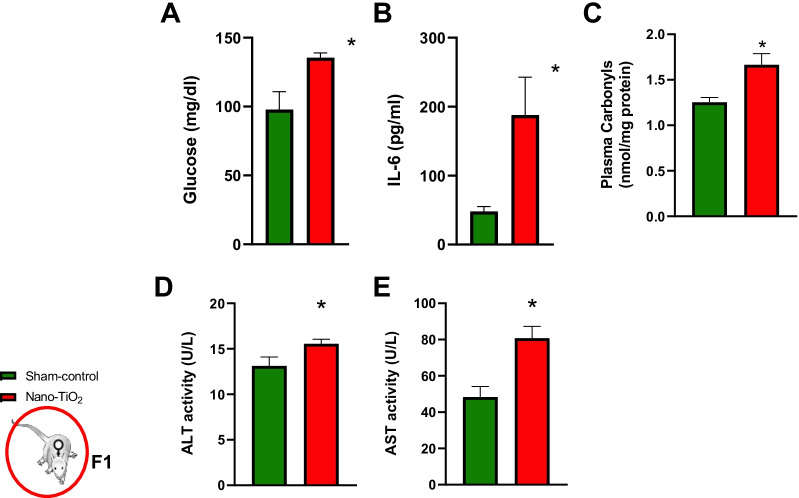
Circulating biological mediators at GD 20 in F1 females. **A** Glucose, **B** Interleukin-6 at GD 20 and **C** plasma carbonyls at GD 20 in F1 dams. n = 6; *p < 0.05 Sham-control group versus nano-TiO_2_-exposed groups

**Fig. 6:**
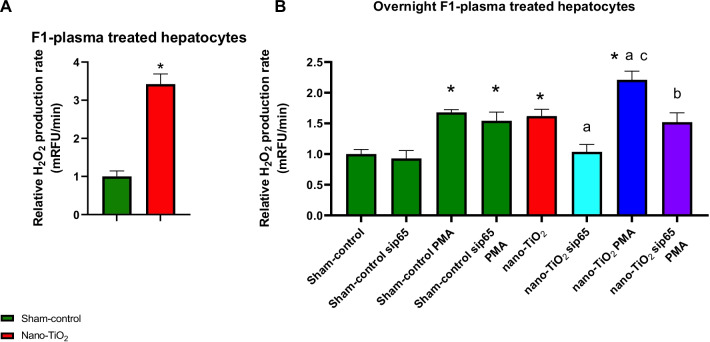
Hydrogen peroxide production rate with silencing RNA. **A** Hepatocytes H_2_O_2_ production capacity detected by coumarin boronic acid following 30-min exposure to 1:2 diluted F1 plasma. **B** Hepatocytes transfected with scrambled or p65 targeted siRNA were exposed overnight to 1:2 diluted F1 plasma then H_2_O_2_ production capacity detected by coumarin boronic acid with or without Phorbol 12-myristate 13-acetate (PMA). n = 6; *p < 0.05 Sham-control group versus nano-TiO_2_-exposed groups

Male offspring, 8 weeks of age, born to mothers exposed to nano-TiO_2_ have higher capacity to produce H_2_O_2_ in liver, aorta (Fig. 4A, B), brain, pancreas, and lung compared to sham-controls (Additional file 1: Figure S1A–C). Western blot analysis showed elevated p-p65 levels in both the aorta and the liver of nano-TiO_2_ offspring (Fig. 4C, D). Additionally, the glutathione antioxidant system is diminished in aorta and liver, presenting with lower total, reduced GSH, and GSH:GSSH ratio (Fig. 4E–J). However, the robust catalase levels in the liver appear unaltered (Fig. 4K). Additionally, in the plasma of F1 dams at GD 20 we detected elevated levels of glucose, IL-6, protein carbonyls, ALT and AST (Fig. 5). Finding elevations of known H_2_O_2_ inducers in the plasma we conducted experiments to determine if plasma factors (collected from F1 offspring at 8 weeks of age; n = 8) could drive H_2_O_2_ production capacity in hepatocytes. First, acute exposure of a hepatic cell line to 1:2 diluted plasma from nano-TiO_2_ F1 induced a threefold increase in H_2_O_2_ production capacity compared to sham-control plasma (Fig. 6A). To assess the impact of these plasma factors on “long term” hepatic redox regulation, cells were incubated with 1:2 diluted plasma overnight with or without siRNA against p65 subunit of NF-κB. Hepatic cells exposed to F1 nano-TiO_2_ plasma exhibited greater H_2_O_2_ production capacity without stimulation. Furthermore, these cells exposed to F1 nano-TiO_2_ plasma had a higher capacity to produce H_2_O_2_ when stimulated with 5 µM PMA. This capacity was abrogated in large part in hepatic cells transfected with 10 nM siRNA against p65 (Fig. 6B). It is clear from this data that redox dysfunction persists into adulthood in pups born to nano-TiO_2_ exposed dams. This clearly demonstrates that circulating factors found within the plasma of adults born to nano-TiO_2_ expose dams induces short- and long-term hepatic redox dysfunction.

### Experiment 2

#### Pup weight, placenta weights and placental efficiency in F2 litters

Litter size (11.53 ± 0.68 pups vs. 9.52 ± 0.57 pups), wet (5.78 ± 0.09 g vs. 4.93 ± 0.47 g) and dry pup weight (Additional file 2: Table S1), and wet placental efficiency (7.51 ± 0.29 vs. 6.48 ± 0.18) were all significantly reduced in F2 litters born to F1 dams with nano-TiO_2_ mothers when compared to F1 dams with sham- control mothers (Table 2). This is of note because despite not being exposed during gestation, dams born to nano-TiO_2_ exposed dams still demonstrated poor gestational and fetal health outcomes at GD 20.

**Table 2:**
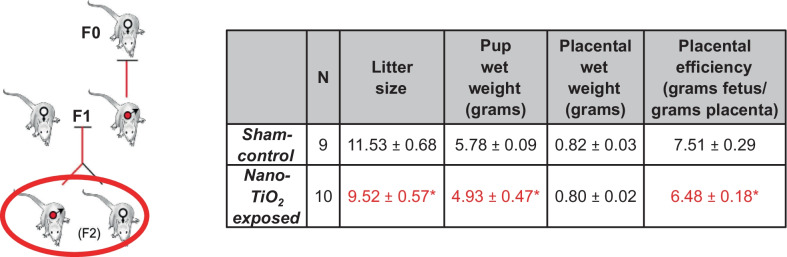
Pup and placental characteristics from F1 dams at GD 20

Pup and placental characteristics in sham-control (N = 9) and nano-TiO_2_ inhalation exposed (N = 10) groups. Values are shown as mean ± SEM.

*p < 0.05 Sham-control group versus nano-TiO_2_-exposed groups.

#### Uterine artery characteristics

Turning our attention to female F1 nano-TiO_2_ offspring, we were interested in looking again at the uterine vasculature at GD 20. Sham-control and nano-TiO_2_-exposed uterine arteries had similar outer diameters (sham-control = 705 ± 51 µm vs. exposed 643 ± 33 µm). However, the inner diameter was significantly smaller in nano-TiO_2_ exposed uterine arteries (502 ± 29 µm) compared to sham-control (591 ± 35 µm) and had increased tone (38 ± 4% vs. 25 ± 2% exposed and sham-control vessels, respectively). (Table 3). These observations point to local vascular dysfunction during gestation that persists in F1 pups born to nano-TiO_2_ exposed mothers.

**Table 3:**
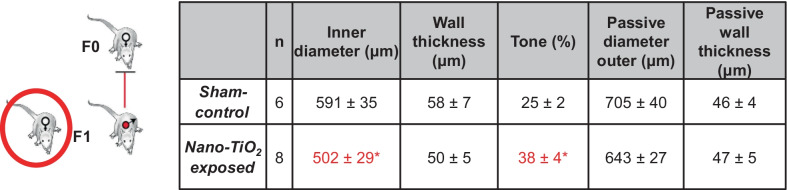
Uterine artery characteristics in F1 dams

Uterine artery characteristics in sham-control (N = 6) and nano-TiO_2_ inhalation exposed (N = 8) groups. Values are shown as mean ± SEM.

*p < 0.05 Sham-control group versus nano-TiO_2_-exposed groups.

#### Vasoreactivity assessments

**Fig. 7:**
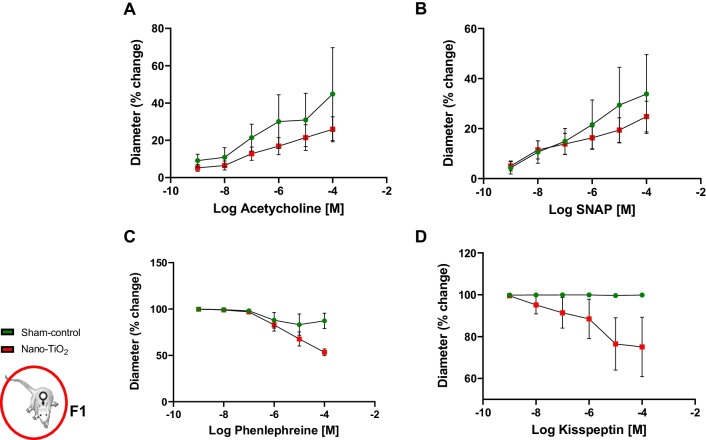
F1 dam uterine artery reactivity at GD 20 using pressure myography. No significant differences were observed between groups when uterine arteries were treated with **A** acetylcholine or **B** SNAP. Increased vasoconstriction was also observed with **C** phenylephrine and **D** kisspeptin at the most concentrated dose (*N* = 6–8). *p < 0.05 Sham-control group versus nano-TiO_2_-exposed groups

**Fig. 8:**
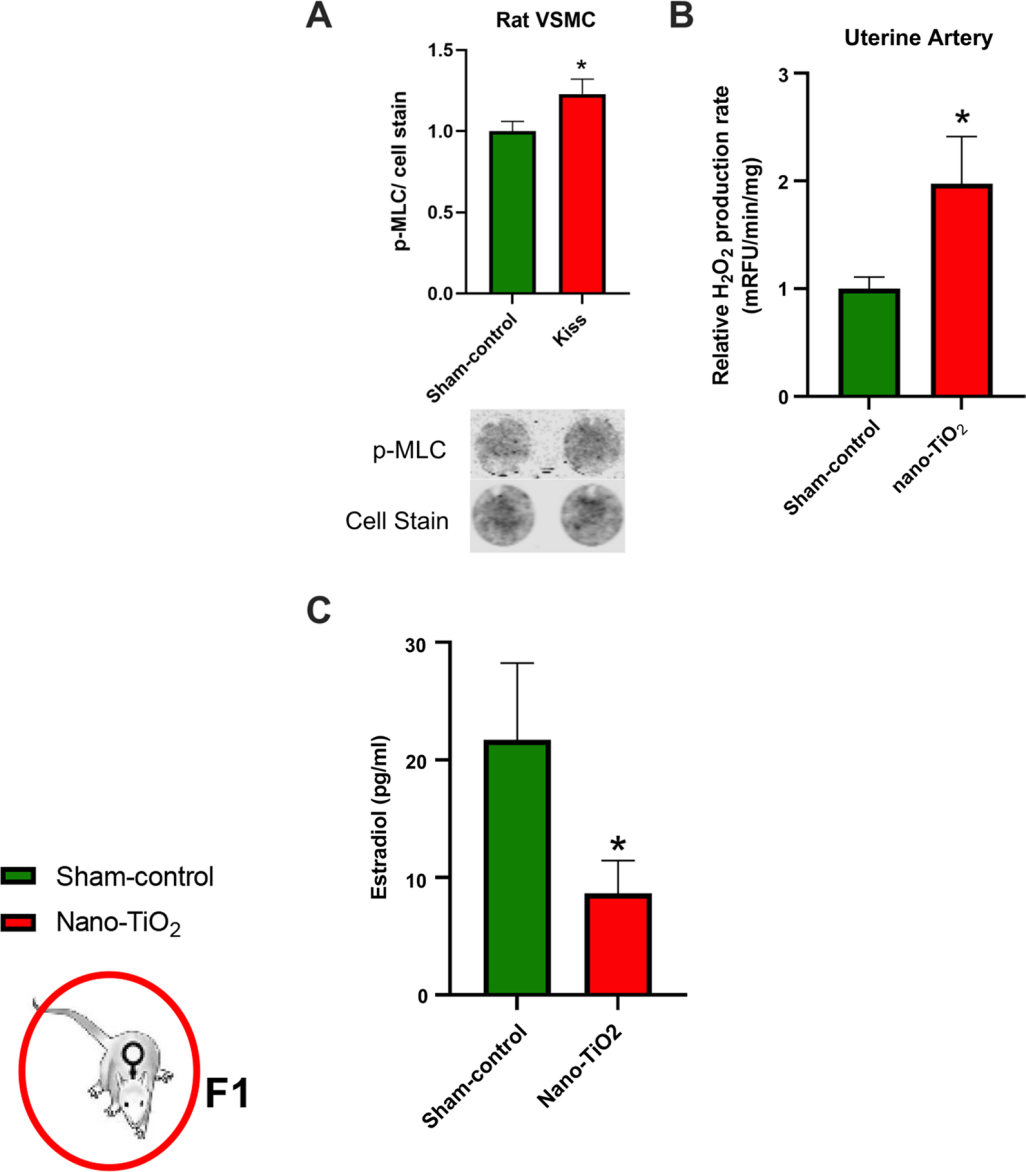
Endocrine and reactive oxygen species disruption in F1 dams at gestational day 20. **A** Kiss treatment of rat aortic smooth muscle cells (RASMC) resulted in a rapid phosphorylation of myosin light chain (MLC) in vascular smooth muscle cells. Representative imagine taken from RASMC cell culture showing rapid phosphorylation of MLC. **B** H_2_O_2_ production capacity rate in isolated uterine arteries. Elevated H_2_O_2_ capacity in F1 TiO_2_ uterine vasculature at GD 20. **C** An ELISA for estradiol was on plasma from F1 females born to sham-control (n = 9) or nano-TiO_2_ dams (n = 12). *p < 0.05 Sham-control group versus nano-TiO_2_-exposed groups

Similar to our observations in uterine arteries from F0 dams that were directly exposed to nano-TiO_2_ during gestation [9], uterine arteries from F1 dams born to mothers exposed to nano-TiO_2_ demonstrated an augmented vasoconstrictor response to Kiss (15.83% overall diameter decrease in nano-TiO_2_ exposed vs. 1.2% in sham-control *p* < 0.05; Fig. 7D). Additionally, F1 uterine arteries from dams born to exposed mothers had an augmented response to PE at the 10^–4^ M dose (Fig. 7C). No other point-to-point differences were seen between groups when vessels were treated with endothelium–dependent or independent dilators (Fig. 7A, B). Mechanistically, we were interested if Kiss directly elicited myosin light chain (MLC) phosphorylation. We found that Kiss treatment of aortic smooth muscle cells resulted in a rapid phosphorylation of MLC (Fig. 8A). In addition, we assessed H_2_O_2_ production capacity in isolated uterine arteries at GD 20 and found elevated H_2_O_2_ capacity in F1 TiO_2_ females (Fig. 8B), which was similar to findings in the aorta of their aged matched male litter mates. These data demonstrate that vascular and redox dysfunction, specifically an increased vasoconstrictor response potentially mediated by increased MLC phosphorylation and elevated uterine artery H_2_O_2_ capacity, during gestation generationally persists in the F1 dams born to nano-TiO_2_ mothers.

#### Assessment of plasma hormones at gestational day 20 in F1 dams

Circulating estrogen was significantly decreased at GD 20 in F1 dams with mothers exposed to nano-TiO_2_ (12.12 ± 3.1 pg/ml) compared to F1 dams with sham-control mothers (29.81 ± 8.8 pg/ml *p* < 0.05) (Fig. 8C). However, there were no differences in circulating levels of FSH, LH, P4 or Kiss (data not shown). This indicates that endocrine disruption may occur across generations, and specifically in this case even though these F1 dams were not directly exposed during their gestations.

## Discussion

These studies provide critical insight into the mechanisms regulating the multigenerational effects of maternal ENM inhalation exposure. We report that elevated H_2_O_2_ production capacity potentially corresponds to upregulation of NF-κB across generations. Furthermore, we provide novel evidence that offspring born to nano-TiO_2_ exposed mothers present with systemic endocrine disruption (estrogen) and alterations in circulating biological mediators (IL-6, glucose, protein carbonyls). This is highlighted by plasma exposed hepatic cells increased H_2_O_2_ production, which is an effect that is mitigated by silencing the p65 subunit of NF-κB. We also observed uterine artery and fetoplacental unit dysfunction at GD 20 in F1 dams born to mothers exposed to nano-TiO_2_ as previously observed in the F0 dams [10]. Taken together these data illustrate hepatic, vascular, and endocrinological dysfunction in the F1 generation due to gestational exposure in their mothers.

Tightly regulated redox signaling is critical in controlling cellular homeostasis. Our data demonstrate that nano-TiO_2_ exposure during gestation leads to systemically upregulated H_2_O_2_ production capacity and diminished GSH defense. Further, our cell culture experiments suggest not only elevated H_2_O_2_ production capacity in a non-challenged state but also a greater H_2_O_2_ production capacity when stimulated by PMA. Considered together, the F1 nano-TiO_2_ generation may be ill equipped to handle physiological challenges such as viral infection dietary challenge or disease. Ultimately, elevated or exaggerated oxidant production in the F1 offspring potentially increases their risk of developing cardio-metabolic pathologies [23, 37–41]. Hepatic oxidant production is linked to numerous pathologies including non-alcoholic fatty liver [42, 43], diabetes [44–46], steatosis [47], and cirrhosis [48], which all in turn increase cardiovascular disease risk [49]. Vascular oxidant production is linked to insulin resistance, atherosclerosis, and peripheral artery disease [50]. Whether or not hepatic vascular dysfunction associated with inhalation exposure during gestation impacts uterine, placental, or fetal vascular health remains to be determined in future studies.

One prominent redox sensitive transcription factor is NF-κB [26]. Our data reflects an increased NF-κB activation in maternal liver of dams exposed to nano-TiO_2_ as well as liver and aorta of their offspring. The observed elevation of IL-6, glucose, and H_2_O_2_ could all contribute to activation of NF-κB [26, 51, 52]. Redox regulation of NF-κB is complex as both activation and inactivation have been detailed. Redox activation of NF-κB can occur at many levels: activation of upstream kinases, inactivation of NF-κB inhibitors, and direct oxidation [53]. Direct oxidation has been reported to decrease the interaction between NF-κB and DNA in vitro [54]. However, nuclear translocation of oxidized NF-κB results in interaction with REF-1, reduction, and increased DNA binding [27], a feature shared by other redox activated transcription factors [55]. While the current study defines the relationship between maternal nano-TiO_2_ inhalation exposure during gestation, H_2_O_2_ and NF-κB, it is very probable that other transcriptional and signaling pathways are also altered. This topic research topic is relatively unexplored and future studies are needed to better understand the redox-mediated regulation of gene expression following inhalation exposure.

NF-κB over activation may in part account for the great capacity to produce oxidants. Indeed, our cell exposure experiments support the notion that NF-κB is important in mediating the upregulation of H_2_O_2_ production capacity induced by F1 nano-TiO_2_ plasma. NF-κB has been shown to regulate a number of oxidant producing enzymes including NADPH oxidases and xanthine oxidase [56–58]. This study shows upregulated NF-κB and H_2_O_2_ production capacity in vascular and hepatic tissue. NF-κB in the vasculature has been linked with increased vasoconstriction [59], remodeling [60], and atherosclerosis [61]. Suggesting offspring of nano-TiO_2_ mothers may be at greater risk of these vascular pathologies as they age or experience a secondary insult, such as consuming a western diet. Future studies will be aimed at understanding the mechanisms of this circular relationship between H_2_O_2_ and NF-κB by way of understanding oxidant sources, NF-κB interaction and gene targets in these tissues.

In addition to NF-κB, exposure to chronically elevated levels of H_2_O_2_ can lead to alterations in multiple signaling pathways regulating cellular function and gene regulation. Evidence exists suggesting H_2_O_2_ directly or indirectly, through thiol antioxidant relay, can modify histones regulating chromatin structure and gene expression [62]. Thus, it is possible the observed elevated H_2_O_2_ production capacity leads to propagation of nano-TiO_2_ impact cross generations through alteration in chromatin structure and gene regulation. Future studies can achieve this by utilizing RNA-Seq and Assay for Transposase-Accessible Chromatin with high throughput sequencing (ATAC-Seq) technologies, as well as 3D nuclear imaging techniques.

Having previously shown that the receptor for Kiss, Kiss1R, is present in the uterine vascular smooth muscle layer [10] we examined estrogen and the vasoreactivity of the uterine artery of F1 dams to Kiss to determine if endocrine-mediated vasoconstriction persists across generations. F1 dams born to mothers exposed to nano-TiO_2_ had significantly reduced circulating estrogen levels compared to F1 dams born to control mothers at GD 20. This is of particular interest because these rats were not exposed during their gestations but still suffered endocrine disruption. Estrogen has long been known to play a critical role in women’s health during gestation specifically regulating uterine and placental blood flow. In specific regards to data presented in this manuscript, estrogen receptor activation is associated with increased antioxidant defense and direct inhibition of NF-κB activation [63–65]. Additionally, oxidant production has been shown to reduce estrogen production and release. This implies a detrimental feed-forward interaction of oxidant-mediated reduction of estrogen and estrogen deficiency-induced upregulation of oxidant signaling following maternal nano-TiO_2_ inhalation exposure.

Uterine arteries from F1 dams born to mothers exposed to nano-TiO_2_ had an elevated tone (38 ± 4% exposed vs 25 ± 2% sham-control). This was characterized anatomically by a smaller luminal diameter, increased wall thickness (Table 3), and physiologically by an assumed vasoconstrictor response to Kiss (Fig. 7). These are similar to observations made in the uterine arteries of dams that were directly exposed to nano-TiO_2_ [9]. We then tested whether Kiss could induce the phosphorylation of MLC utilizing rat aortic smooth muscle cells. We found that in culture, phosphorylation of MLC increased modestly following 10 min of Kiss exposure. This result suggests a potential mechanism of Kiss induced vasoconstriction in pressurized uterine arteries. Oxidant production and redox signaling have been previously reported to alter vascular tone and remodeling [66]. Thus, we assessed the H_2_O_2_ production capacity of isolated uterine arteries and found F1 nano-TiO_2_ uterine arteries had a greater H_2_O_2_ production capacity. This is further evidence of redox dysregulation in the F1 generation of mothers exposed to nano-TiO_2_. We speculate that redox dysregulation contributes to the increased vasoconstrictive Kiss response as well as the vascular wall thickening.

Collectively, these results suggest decreased blood flow to fetoplacental units in F0 dams exposed to nano-TiO_2_ [16] and their F1 offspring during gestation (current data). Indeed, similar to what was previously reported in nano-TiO_2_ F1 pups at GD 20, F2 pups measured significantly smaller than air exposed F2 pups. These results indicate that a single period of gestational exposure affects the health of at least two generations and has long-term consequences in male and female adults born to exposed mothers. Propagation of these multigenerational health effects are likely rooted in changes to chromatin structure and gene regulation. Critical insight into mechanisms of nano-TiO_2_-induced multigenerational dysfunction could be gained through sequencing and “omic” style approaches.

One limitation of the current study is that the plasma samples for endocrine analysis were only taken a single time point during gestation. Despite estrogen disruption, other key reproductive hormones such as P4, LH, FSH, and Kiss were unchanged at GD 20, which is similar to what we observed in our previous study in samples taken from dams that were directly exposed [10]. Sampling at different key time points such as implantation or placentation could reveal disruption of other gestational and metabolic factors.

## Conclusion

**Fig. 9:**
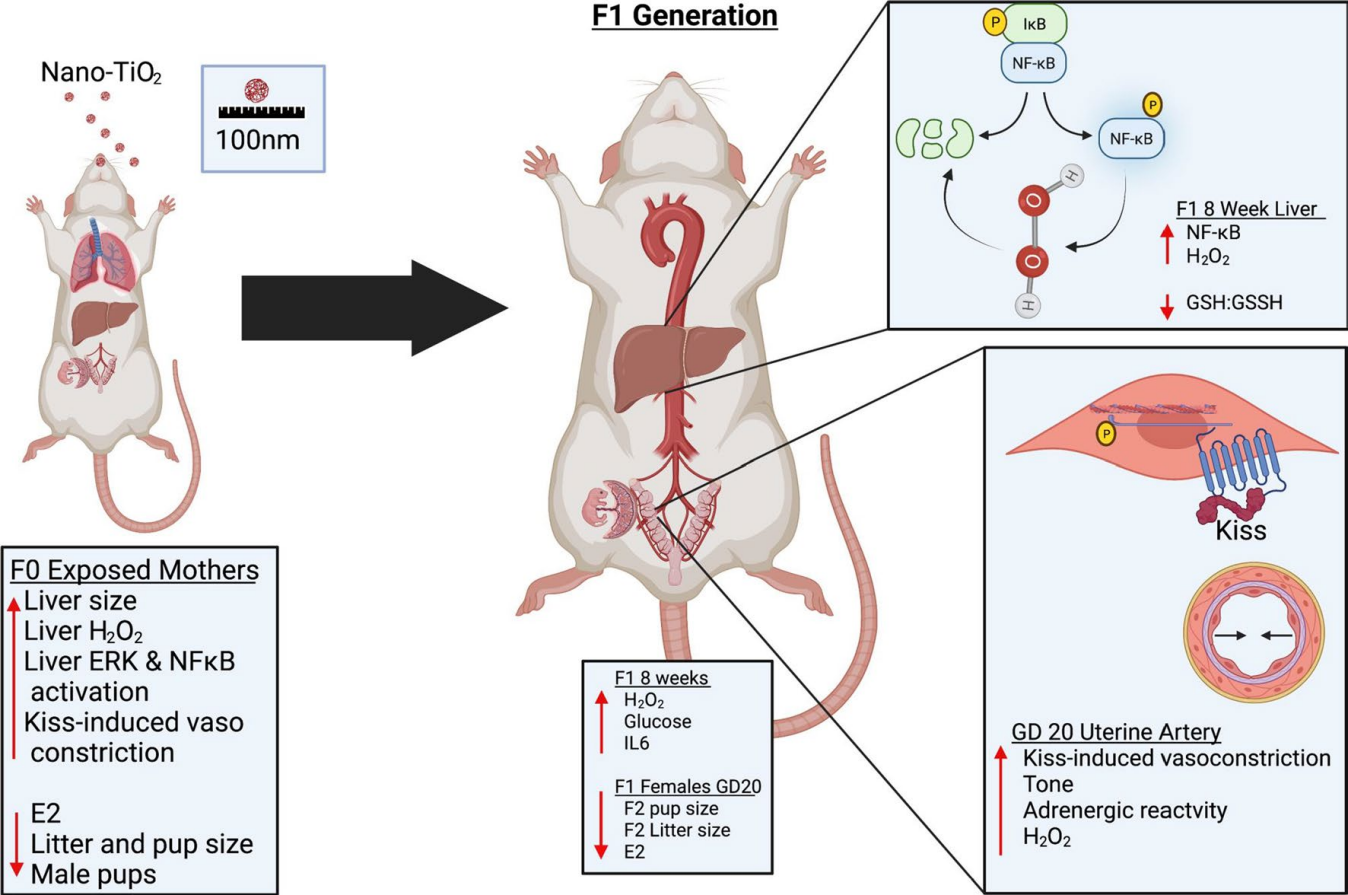
Summary of the vascular and redox dysfunction in the F1 generations after maternal nano-TIO_2_ inhalation exposure. Direct hepatic and reproductive effects on the F0 dams is seen on the left. Endocrine and hepatic perturbations were observed in the F1 males as adults at 8 weeks of age, as well as in the females at GD 20. Additionally, kisspeptin increased vasoconstriction in the uterine artery at GD 20 in F1 females along with increased adrenergic response, H_2_O_2_ production, and tone. *p < 0.05 Sham-control group versus nano-TiO_2_-exposed groups

In conclusion, this manuscript provides novel evidence that maternal ENM inhalation exposure results in multigenerational effects. Our data indicate systemic upregulation of H_2_O_2_ production capacity and NF-κB activation in F0 dams and their offspring (Fig. 9). We speculate that this increases the susceptibility of the F1 generation to various pathologies in adulthood. A focused probe of the F1 uterine vasculature at GD 20 revealed altered gestational function similar to the F0 dams, again resulting in undersized offspring in the F2 generation. The extent of this dysregulation and how long the dysfunction persists and the mechanism by which these outcomes are perpetuated beyond the F1 generation remains a topic ripe for exploration.

## Materials and methods

### Animal model

Female, Sprague–Dawley (SD rats) were purchased from Hilltop Laboratories (Scottdale, PA), and housed in an Assessment and Accreditation of Laboratory Animal Care (AAALAC) approved facility at West Virginia University (WVU) under a regulated temperature and 12:12 h light–dark cycle. Rats were randomly assigned to either the sham-control or nano-TiO_2_ exposure groups and acclimated for 48–72-h before mating. Rats had ad libitum access to food and water throughout the acclimation period. To increase the likelihood of viable progeny, pregnant rats were exposed to nano-TiO_2_ aerosols as described below on or after implantation gestational day 10 (GD 10) as prior indications of inhalation exposure results in near to total loss of pregnancy [15]. Weights of the pregnant dams were recorded weekly. Dams were allowed to deliver pups naturally. Pups were housed with dams for 21 days and weaned based on sex. Once female pups (F1 females) reached sexual maturity (6–8 weeks of age) they were mated to control males to establish pregnancy and euthanized on GD 20. All procedures were approved by the Institutional Animal Care and Use Committee of West Virginia University.

### Engineered nanomaterial

Nano-TiO_2_ powder was obtained from Evonik (Aeroxide TiO_2_, Parsippany, NJ). It is a mixture composed of anatase (80%) and rutile (20%) TiO_2_. Particle characteristics have been determined including the primary particle size (21 nm), the specific surface area (48.08 m/g), and the Zeta potential (− 56.6 mV) [7].

Aerosol size distributions were determined in the exposure chamber while the target mass concentration was being maintained at 12 ± 0.13 mg/m^3^ with: (1) a high-resolution electrical low-pressure impactor (ELPI+; Dekati, Tampere, Finland), (2) a scanning particle mobility sizer (SMPS 3938; TSI Inc., St. Paul, MN), and (3) an aerodynamic particle sizer (APS 3321; TSI Inc., St. Paul, MN), and a Nano Micro-Orifice Uniform Deposit Impactor (MOUDI 115R, MSP Corp, Shoreview, MN).

### Inhalation exposure

Nano-TiO_2_ aerosols were generated using a high-pressure acoustical generator (HPAG, IEStechno, Morgantown, WV). The output of the generator was fed into a Venturi pump (JS-60 M, Vaccon, Medway, MA) which further de-agglomerated the particles. The nano-TiO_2_ aerosol/air mix then entered the whole-body exposure chamber. A personal DataRAM (pDR-1500; Thermo Environmental Instruments Inc., Franklin, MA) was utilized to sample the exposure chamber air to determine the aerosol mass concentration in real-time. Feedback loops within the software automatically adjusted the acoustic energy to maintain a stable mass concentration during the exposure. Gravimetric measurements were conducted on Teflon filters concurrently with the DataRAM measurements to obtain a calibration factor. The gravimetric measurements were also conducted during each exposure to calculate the mass concentration measurements reported in the study. Bedding material soaked with water was used in the exposure chamber to maintain humidity (30–70%) during exposures. Sham-control animals were exposed to HEPA filtered air only with similar temperature and humidity chamber conditions.

Inhalation exposures in F0 dams lasted for 6 days after GD 10 to decrease animal stress. The pregnant rats were exposed to an average target concentration of 12 mg/m^3^. This concentration was chosen to match our previous late gestation inhalation exposure studies [14, 15] To estimate lung dose with nano-TiO_2_ aerosols [13] we used the equation: D = F⋅V⋅C⋅T, where F is the deposition fraction (10%), V is the minute ventilation (208.3 cc), C equals the mass concentration (mg/m^3^), and T equals the exposure duration (minutes) [67]. This exposure paradigm (12 mg/m^3^, 6 h/exposure, 6 days) produced an estimated target lung dose of 525 ± 16 µg with the last exposure conducted 24 h prior to sacrifice and experimentation. These calculations represent total lung deposition and do not account for clearance (MPPD Software v 2.11, Arlington, VA).

### Enzyme linked immunosorbent assay (ELISA)

F1 Dams were anesthetized with isoflurane gas (5% induction, 2–3.5% maintenance). The animals were placed on a heating pad to maintain a 37 °C rectal temperature. The trachea was intubated to ensure an open airway and the right carotid artery was cannulated to allow for blood sampling of ~ 3 mL. Plasma and serum were collected on GD 20 and ELISAs were performed for estrogen, progesterone (P4), luteinizing hormone (LH), and follicle stimulating hormone (FSH). All kits were performed according to the recommendation of the manufacturer (CalBiotech, Spring Valley, CA). Additionally, plasma was collected at 8 weeks of age in F1 males and females was assayed for IL-6 (R&D Systems, Minneapolis, MN), glucose (Cayman, Ann Arbor, MI), and plasma protein carbonyls (Abcam, Cambridge, MA). Enzymatic activity for ALT and AST were measured with plasma from F0 dams at GD 20 (n = 8) and F1 at 8 weeks of age (n = 8) samples following the manufacturer’s instructions (Cayman, Ann Arbor, MI).

### Gestational outcomes in F0 and F1 dams

Liter size, sex ratio, implantation sites, and uterine weights were recorded in F0 and F1 dams. Once trunk blood was collected from F1 dams, F2 pups and placenta were carefully dissected away from the uterine wall and weighed individually immediately after sacrifice (wet weight) and after desiccation (dry weight). F2 pup and placental weights were measured to calculate placental efficiency (grams fetus/gram placental).

### Cell culture

AML12 hepatocytes were grown in DMEM/F12 (ThermoFisher Scientific, Waltham, MA) media supplemented with dexamethasone, (ThermoFisher Scientific, Waltham, MA) growth supplement (ThermoFisher Scientific, Waltham, MA), and 10% FBS (ThermoFisher Scientific, Waltham, MA). For plasma exposure AML12 cells were seeded in a clear bottom black sided 96-well plate (ThermoFisher Scientific, Waltham, MA) and allowed to adhere for 24 h. At ~ 90% confluency the media was replaced with plasma samples mixed with 2 parts culture media and incubated for 1 h prior to H_2_O_2_ measurements. In other experiments AML12 cells were reverse transfected using lipofectamine 3000 with 10 nM of either scrambled or p65 targeted siRNA (ThermoFisher Scientific, Waltham, MA) when plated in the 96-well plates. Cells were grown in these conditions for 48 h. Then media was replaced with plasma (collected from F1 offspring at 8 weeks of age; n = 8) spiked culture media as above and left overnight before assessing H_2_O_2_ measurements.

Rat Aortic smooth muscle cells (RASMC) were grown in Lonza smooth muscle complete media (Lonza, Basel, Switzerland). For experiments RASMC were plated in 6-well or clear bottom black sided 96-well plate. Cells were then exposed to 1 ng/ml of Kiss for the times indicated (0, 5, or 10 min) and 96-well plates fixed for in-cell western.

### Coumarin boronic acid assay

Coumarin Boronic acid (CBA) was conducted as previously published [55, 68–71]. Tissue was homogenized in PBS (Fisher Scientific, Waltham, MA) containing a protease and phosphatase cocktail (Thermofisher Scientific, Waltham, MA). 10 µl of equal concentration sample homogenate were loaded into a black sided 96 well plate along with 90 µl of CBA buffer (containing PBS, L-Name (Sigma-Aldrich, St. Louis, MO), Taurine (Sigma-Aldrich, St. Louis, MO), and 500 µM CBA probe (Cayman, Ann Arbor, MI) ± 1 KU catalase (Sigma-Aldrich, St. Louis, MO). For 96-well plates cell culture media was removed and 100 µl of CBA buffer (again containing 500 µM of CBA probe) was added ± 1 KU catalase. Plates were then run in a 37 °C plate reader and fluorescence measured (ex: 350, em: 450) every minute over 2 h. Signal from the negative control catalase wells were subtracted out from the sample wells and only the catalase inhibitable signal was analyzed. The rate of the relative fluorescent units per minute was calculated for all samples and fold change from control treatment calculated.

### Glutathione assay

Samples were prepared and according to the kit protocol (ab239709, Abcam, Cambridge, MA). In brief, aorta and liver were homogenized in assay buffer and each sample was run with and without glutathione reductase to measure total GSH, reduced GSH, and calculate the oxidized to reduced ratio.

### Western blot

Medial lobe liver sections were snap frozen at the time of euthanasia. Liver tissue was homogenized in RIPA buffer containing a protease and phosphatase cocktail (Thermofisher, Waltham, MA). Samples were then prepared with laemmli sample buffer and β-mercaptoethanol at a 4 µg/µl concentration and 32 µg of total protein run down each lane of a 4–20% gradient gel (Biorad, Hercules, California) at 70 V. Samples were then transferred to 0.45 µm nitrocellulose membranes, dried, reconstituted with _ddi_H_2_O, blocked with LiCor (Lincoln, Nebraska) TBS blocking buffer, and incubated in primary antibody overnight at 4 °C. Primary antibodies include β-actin (Santa Cruz Biotechnology, Dallas, Texas), phospho myosin light chain (Thr18/Ser19) (Cell Signaling Technologies, Danvers, MA), phospho and total p65 (Cell Signaling Technologies, Danvers, MA), phospho and total ERK 1/2 (Cell Signaling Technologies, Danvers, MA), and catalase (Abcam, Cambridge MA). Membranes were then washed with TBS containing 1% tween (TBST), incubated in Licor near-infrared secondary antibodies for 1 h at room temperature, washed again with TBST, and finally imaged with the LiCor Odyssey Clx. Densitometry analysis was conducted in Image-J (National Institutes of Health, Bethesda, Maryland) with phosphor-signal normalized to total-signal and fold change calculated from control.

### Isolated microvessel protocol (pressure myography)

After the placenta and pups were removed, uteri were placed in a dissecting dish with physiological salt solution (PSS), as previously described [72], and maintained at 4 °C. A uterine artery segment was isolated, removed and transferred to a vessel chamber (Living Systems Instrumentation, Burlington, VT) containing fresh oxygenated PSS, cannulated with glass pipettes, and secured using nylon suture (11-0 ophthalmic, Alcon, U.K.). Arteries were extended to their in-situ length, pressurized to 60 mm Hg with PSS, superfused with warmed.

(37 °C) oxygenated PSS at a rate of 10 mL/min, and allowed to develop spontaneous tone. Internal and external arteriolar diameters were measured using video calipers (Colorado Video, Boulder, CO).

### Uterine vasculature reactivity

Uterine arteries were allowed to develop spontaneous tone, defined as the degree of constriction experienced by a blood vessel relative to its maximally dilated state. Vascular tone ranges from 0% (maximally dilated) to 100% (maximal constriction). Vessels with a spontaneous tone ≥ 20% less than initial tone were included in this study. After equilibration, parameters of arterial vasoreactivity were analyzed. Vessels that did not develop sufficient spontaneous tone were not included in the data analysis.

### Assessment of vasoreactivity

Arteries were exposed to increasing concentrations of phenylephrine (PE: 10^−9^ to 10^−4^ M), acetylcholine (ACh: 10^−9^ to 10^−4^ M), sodium nitroprusside (SNP: 10^−9^ to 10^−4^ M) and kisspeptin-10 (Kiss: 10^−9^ to 10^−4^ M), which were each added separately to the bath. The steady state diameter of the vessel was recorded for at least 2 min after each dose. After each dose curve was completed, the vessel bath was exchanged to remove excess chemicals by carefully removing the superfusate and replacing it with fresh warmed oxygenated PSS. After all experimental treatments were complete, the PSS was replaced with Ca^2+^-free PSS until maximum passive diameter was established.

## Pressure myography calculations

Spontaneous tone was calculated by the following equation:

$$Spontaneous\,tone\left( \% \right) = \left\{ {\frac{{\left( {Dm - Di} \right)}}{{Di}}} \right\}{\text{ }} \times 100$$

where D_m_ is the maximal diameter and D_i_ is the initial steady state diameter recorded prior to the experiment. Active responses to pressure were normalized to the maximal diameter using the following formula:

$$Normalized\,diameter = Dss{\text{/}}Dm$$

where D_SS_ is the steady state diameter recorded during each pressure change. The experimental responses to ACh, PE, and SNP are expressed using the following equation:

$$Diameter\left( {percent\,maximal\,diameter} \right) = \left\{ {\frac{{\left( {Dss - Dcon} \right)}}{{(Dm - Dcon)}}} \right\} \times 100$$

where D_con_ is the control diameter recorded prior to the dose curve, D_SS_ is the steady state diameter at each dose of the curve. The experimental response to PE is expressed using the following equation:

$$Diameter\left( {percent\,maximal\,diameter} \right) = \left\{ {\frac{{\left( {Dcon - Dss} \right)}}{{(Dcon)}}} \right\} \times 100$$

Wall thickness (WT) was calculated from the measurement of both inner (ID) and outer (OD) steady state arteriolar diameters at the end of the Ca^2+^ free wash using the following equation:

$$WT = (OD - D){\text{/}}2.$$

### Statistics

Data are expressed as means ± standard error pf the mean. Point-to-point differences in the dose response curves were evaluated using two-way repeated measures analysis of variance (ANOVA) with a Tukey`s *post-hoc* analysis when significance was found. The animal characteristics, vessel characteristics and hepatocytes transfected with siRNA were analyzed using a one-way ANOVA with a Tukey *post-hoc* analysis when significance was found. Student’s t-test was utilized for comparison of two groups (i.e. liver weights). All statistical analysis was completed with Graph Pad Prism (San Diego, CA) Significance was set at *p* < 0.05, N is the number of animals per group, n is the number of vessels per group.

## Supplementary Information

**Additional file 1. Figure S1: **Hydrogen peroxide production rate in additional tissues from F1 males at 8 weeks of age. H_2_O_2_ production capacity detected by coumarin boronic acid in other F1 tissue A) pancreas, B) lung, C) brain. n=4-20. *, p < 0.05 Sham-control group versus nano-TiO_2_-exposed groups. Hepatocytes transfected with scrambled or p65 targeted siRNA were exposed overnight to 1:2 diluted F1 plasma then phosphorylation status determined (n=6); *, p < 0.05 Sham-control group versus nano-TiO_2_-exposed groups.

**Additional file 2. Table S1: **Pup and Placental Characteristics from F1 Dams. Pup and placental characteristics in sham-control (N = 9) and nano-TiO_2_ inhalation exposed (N = 10) groups. Values are shown as mean ± SEM. P ≤ 0.05, * Sham control group vs. nano-TiO_2_ exposed groups.
