# Supplementary figures and images for: Nano-titanium dioxide inhalation exposure during gestation drives redox dysregulation and vascular dysfunction across generations
Source: Part Fibre Toxicol. 2022 Mar 9;19:18. doi: 10.1186/s12989-022-00457-y (PMC8905816; doi:10.1186/s12989-022-00457-y)

Supplemental Figure 1

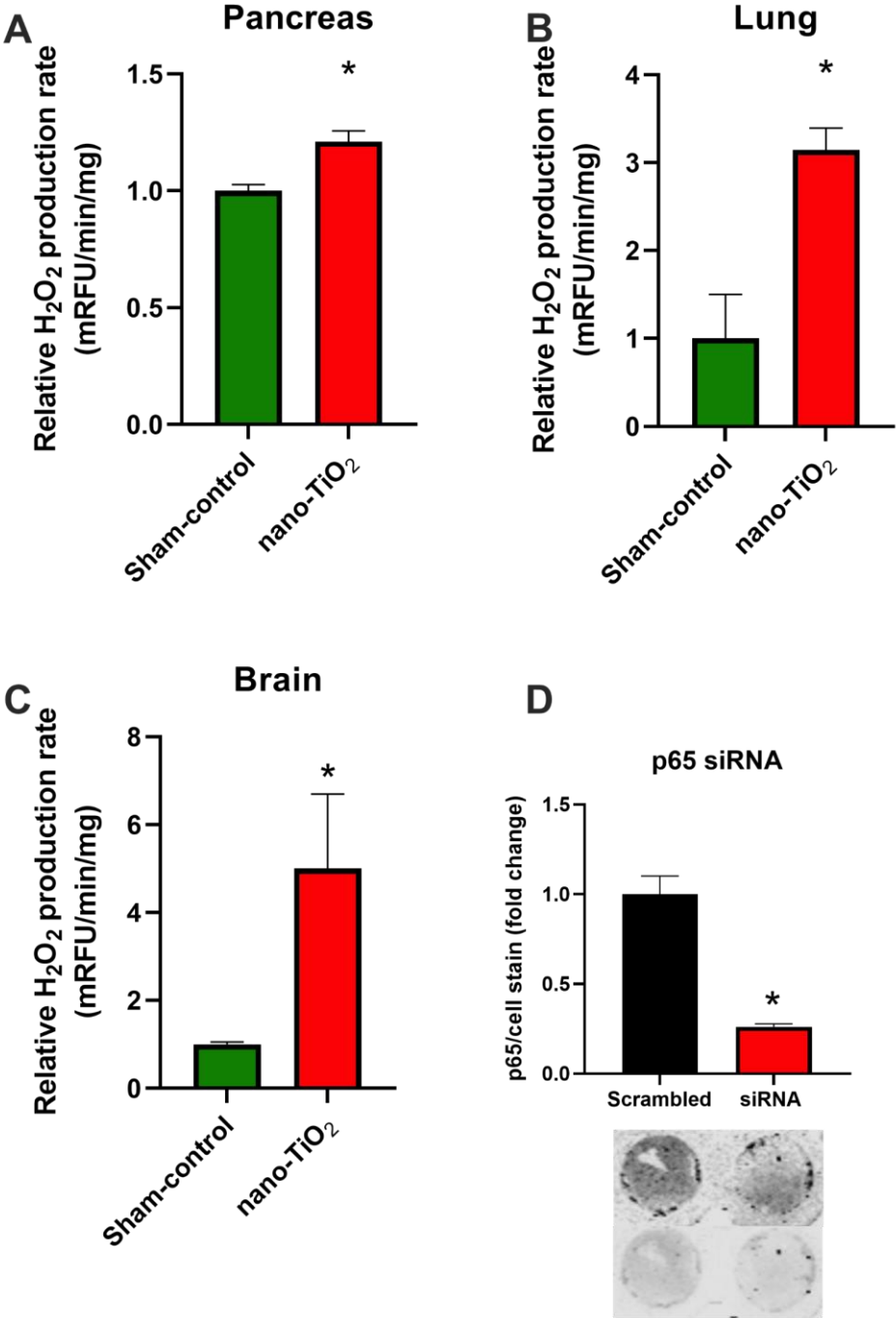

Sham-control  
Nano-TiO<sub>2</sub>

Supplement: Supplementary file 1 — Additional file 1. Figure S1: Hydrogen peroxide production rate in additional tissues from F1 males at 8 weeks of age. H2O2 production capacity detected by coumarin boronic acid in other F1 tissue A) pancreas, B) lung, C) brain. n=4-20. *, p < 0.05 Sham-control group versus nano-TiO2-exposed groups. Hepatocytes transfected with scrambled or p65 targeted siRNA were exposed overnight to 1:2 diluted F1 plasma then phosphorylation status determined (n=6); *, p < 0.05 Sham-control group versus nano-TiO2-exposed groups. [file 12989_2022_457_MOESM1_ESM.pdf]
