# Supplementary material for: Nano-titanium dioxide inhalation exposure during gestation drives redox dysregulation and vascular dysfunction across generations
Source: Part Fibre Toxicol. 2022 Mar 9;19:18. doi: 10.1186/s12989-022-00457-y (PMC8905816; doi:10.1186/s12989-022-00457-y)
Supplement: Supplementary file 2 — Additional file 2. Table S1: Pup and Placental Characteristics from F1 Dams. Pup and placental characteristics in sham-control (N = 9) and nano-TiO2 inhalation exposed (N = 10) groups. Values are shown as mean ± SEM. P ≤ 0.05, * Sham control group vs. nano-TiO2 exposed groups. [file 12989_2022_457_MOESM2_ESM.pdf]

Supplemental Table 1

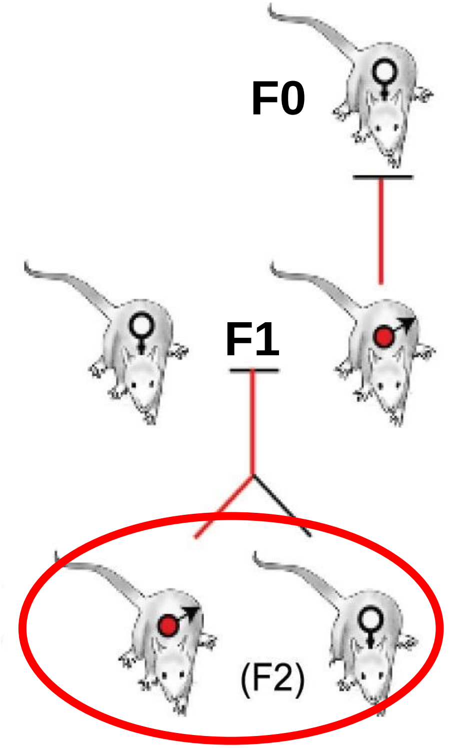

	N	Litter Size	Pup Dry Weight (grams)	Placenta Dry Weight (grams)	Placental Efficiency (grams fetus/ grams placenta)
Sham-Control	9	11.53 ± 0.68	0.77 ± 0.02	0.16 ± 0.01	5.97 ± 0.34
Nano-TiO_2 Exposed	10	9.52 ± 0.57*	0.69 ± 0.02*	0.14 ± 0.01	5.92 ± 0.43

Pup and Placental Characteristics from F1 Dams. Pup and placental characteristics in sham-control (N = 9) and nano-TiO_2 inhalation exposed (N = 10) groups. Values are shown as mean ± SEM. P ≤ 0.05, * Sham control group vs. nano-TiO_2 exposed groups.
